# Natural variables separate the endemic areas of *Clonorchis sinensis* and *Opisthorchis viverrini* along a continuous, straight zone in Southeast Asia

**DOI:** 10.1186/s40249-024-01191-7

**Published:** 2024-03-12

**Authors:** Jin-Xin Zheng, Hui-Hui Zhu, Shang Xia, Men‐Bao Qian, Hung Manh Nguyen, Banchob Sripa, Somphou Sayasone, Virak Khieu, Robert Bergquist, Xiao-Nong Zhou

**Affiliations:** 1 School of Global Health, Chinese Center for Tropical Diseases Research, Shanghai Jiao Tong University School of Medicine, Shanghai, 20025 China; 2 One Health Center, Shanghai Jiao Tong University, The University of Edinburgh, Shanghai, 20025 China; 3 National Institute of Parasitic Diseases at Chinese Center for Disease Control and Prevention (Chinese Center for Tropical Diseases Research), NHC Key Laboratory of Parasites and Vectors Biology, WHO Collaborating Centre for Tropical Diseases, National Center for International Research on Tropical Diseases, Shanghai, 200025 China; 4 Institute of Ecology and Biological Resources, Graduate University of Science and Technology, Vietnam Academy of Science and Technology, 18 Hoang Quoc Viet Street, Hanoi, Vietnam; 5 WHO Collaborating Centre for Research and Control of Opisthorchiasis (Southeast Asian Liver Fluke Disease), Tropical Disease Research Laboratory, Department of Tropical Medicine, Faculty of Medicine, Khon Kaen University, 123 Mittraparb Road, Khon Kaen, 40002 Thailand; 6 Lao Tropical and Public Health Institute, Ministry of Health, Vientiane, Lao PDR; 7 National Centre for Parasitology, Entomology and Malaria Control, Ministry of Health, Phnom Penh, Cambodia; 8 Ingerod, Brastad, Sweden (formerly at the UNICEF/UNDP/World Bank/WHO Special Programme for Research and Training in Tropical Diseases), World Health Organization, Geneva, Switzerland

**Keywords:** Liver fluke, Clonorchiasis, Opisthorchiasis, *Clonorchis sinensis*, *Opisthorchis viverrine*, Southeast Asia, Machine learning, Ecological study

## Abstract

**Background:**

Clonorchiasis and opisthorchiasis, caused by the liver flukes *Clonorchis sinensis* and *Opisthorchis viverrini* respectively, represent significant neglected tropical diseases (NTDs) in Asia. The co-existence of these pathogens in overlapping regions complicates effective disease control strategies. This study aimed to clarify the distribution and interaction of these diseases within Southeast Asia.

**Methods:**

We systematically collated occurrence records of human clonorchiasis (*n* = 1809) and opisthorchiasis (*n* = 731) across the Southeast Asia countries. Utilizing species distribution models incorporating environmental and climatic data, coupled machine learning algorithms with boosted regression trees, we predicted and distinguished endemic areas for each fluke species. Machine learning techniques, including geospatial analysis, were employed to delineate the boundaries between these flukes.

**Results:**

Our analysis revealed that the endemic range of *C. sinensis* and *O. viverrini* in Southeast Asia primarily spans across part of China, Vietnam, Thailand, Laos, and Cambodia. During the period from 2000 to 2018, we identified *C. sinensis* infections in 84 distinct locations, predominantly in southern China (Guangxi Zhuang Autonomous Region) and northern Vietnam. In a stark contrast, *O. viverrini* was more widely distributed, with infections documented in 721 locations across Thailand, Laos, Cambodia, and Vietnam. Critical environmental determinants were quantitatively analyzed, revealing annual mean temperatures ranging between 14 and 20 °C in clonorchiasis-endemic areas and 24–30 °C in opisthorchiasis regions (*P* < 0.05). The machine learning model effectively mapped a distinct demarcation zone, demonstrating a clear separation between the endemic areas of these two liver flukes with AUC from 0.9 to1. The study in Vietnam delineates the coexistence and geographical boundaries of *C. sinensis* and *O. viverrini*, revealing distinct endemic zones and a transitional area where both liver fluke species overlap.

**Conclusions:**

Our findings highlight the critical role of specific climatic and environmental factors in influencing the geographical distribution of *C. sinensis* and *O. viverrini*. This spatial delineation offers valuable insights for integrated surveillance and control strategies, particularly in regions with sympatric transmission. The results underscore the need for tailored interventions, considering regional epidemiological variations. Future collaborations integrating eco-epidemiology, molecular epidemiology, and parasitology are essential to further elucidate the complex interplay of liver fluke distributions in Asia.

**Supplementary Information:**

The online version contains supplementary material available at 10.1186/s40249-024-01191-7.

## Background

The prevalence and infection rates of liver fluke diseases are high across Asian regions, particularly notable in the Mekong River Basin. Those diseases caused by *Clonorchis sinensis* and *Opisthorchis viverrini* are highly prevalent food-borne trematodiasis (FBTs) [1, 2]. Extensive research indicates that clonorchiasis is widespread in parts of Russia, Republic of Korea, southern and northeastern China, extending its endemicity to the northern provinces of Vietnam, exhibiting localized epidemics [3]. In contrast, opisthorchiasis primarily affects the lower Mekong regions, including Thailand, Laos, Cambodia, and the central and southern provinces of Vietnam [4]. Notably, Vietnam is the only country where both types of human liver fluke infections co-exist [5, 6]. This co-endemicity presents unique public health challenges and necessitates targeted intervention strategies to effectively address the burden of these parasitic infections.

First documented in Vietnam in 1887, *C. sinensis* infection was followed by the discovery of *O. viverrini* transmission in central Vietnamese provinces in 1994 [7]. A 1992 epidemiological survey in Phu Yen Province in central Vietnam found *C. sinensis* prevalence ranging from 23.5 to 31.0% in northern Nam Dinh Province, with *O. viverrini* prevalence up to 43.5% in males and 29.4% in females, concentrated among 40–59-year-olds [8]. Subsequently, reports of both liver fluke infections have accumulated in Vietnam. As the only country endemic for both species, Vietnam provides an opportunity to elucidate the interface between their geographic distributions from an epidemiological perspective [9].

Whereas *C. sinensis* is distributed across parts of Russia, Republic of Korea, parts of China and northern Vietnam, *O. viverrini* is concentrated in the lower Mekong River Basin of Thailand, Laos, Cambodia, and central-southern Vietnam [9]. Co-endemicity of both fluke species has been uniquely observed in Vietnam, but early epidemiological data are limited by diagnostic constraints [10]. *C. sinensis* was initially reported from northern Vietnamese provinces only, while *O. viverrini* was restricted to central endemic foci of Vietnam. Although *O. viverrini* has been reported in cats in southern Vietnam, no human cases were documented [11]. Diagnosis, based on microscopic egg morphology in the 1970s–1980s likely resulted in substantial overestimates of national clonorchiasis prevalence, as other intestinal trematode eggs may have been misclassified as those of *C. sinensis*. Updated epidemiological assessments using accurate diagnostics are needed to delineate the endemic boundaries and disease burden posed by each liver fluke species across different regions, particularly in Vietnam.

The life cycles of *C. sinensis* and *O. viverrini* are similar, with humans and some other mammals (pigs, cats, dogs and rodents) as the definite hosts and freshwater snails and fish as two intermediate hosts following each other in that order [12]. Human infections of both flukes are acquired by consuming undercooked freshwater fish harboring infective metacercariae. However, the first intermediate hosts differ between the species. The spatial distribution of the two fluke infections is heavily influenced by the presence of populations of susceptible snail species and suitable environmental conditions [13, 14]. Elucidating the intermediate host profiles and environmental limits of each liver fluke species is imperative to understand their endemic boundaries and opportunities for sustained disease control.

It remains unclear whether clear boundaries exist in the endemic distributions of the two parasites, and which factors that have resulted in their segregated geographic patterns[5]. Predicted distribution maps for clonorchiasis and opisthorchiasis exist, but a proper, integrated analysis investigating the geographic boundaries between the two trematodiases has yet to be conducted. Defining the specific ecologic limits and spatial overlap of these two helminths is imperative to devise integrated control strategies that account for their sympatry across certain endemic regions. A comprehensive approach combining predictive mapping and delineation of the niche boundaries would provide novel insights into their distinct epidemiology in Asia.

The application of machine learning methods for disease distribution prediction represents a major focus in this field. Such approaches primarily address the classification problem of delineating disease ranges based on input variable patterns using algorithms that identify relationships within data to categorize outcome or input variables[15]. Machine learning techniques comprise supervised learning, in which models are trained on known input–output variable pairs to predict outcomes for new inputs (e.g. logistic regression), and unsupervised learning, which uncovers inherent structure within existing data to inform clustering[16]. Given compiled databases of geolocated *C. sinensis* and *O. viverrini* human infection records across the Mekong River Basin, we applied machine learning models to elucidate the niche boundaries between the two liver flukes and characterize key determinants of their divergent endemic patterns. Since algorithms trained on ecological and social data can provide novel insights unavailable from traditional statistical approaches, we applied such techniques for comparative predictive mapping of clonorchiasis and opisthorchiasis distributions. By integrating epidemiological perspectives from the literature with computational modelling techniques, we aimed to elucidate the complex and divergent ecology of these two liver fluke species. The primary aim is to compile location-specific cases of infections to input into ecological niche modelling by machine learning techniques, aiming to map transmission patterns rather than quantify infection prevalence rates. Then secondary aim is to document infection risk factors, identifying potential influences on the models to understand the relationship between environmental variables and geo-locations. This comprehensive approach allows for a detailed comparison of environmental conditions that support the sustenance of these parasites in endemic areas.

## Methods

### Study design

We conducted an integrative modelling study using a mix of data sources to map the niche boundaries and model the divergent epidemiology of clonorchiasis and opisthorchiasis in Asia. The integration of multiple data modalities aimed to provide novel insights into the differing ecology and transmission dynamics of these two liver fluke infections. The modelling framework incorporated three main components: (1) a systematic literature review of prior epidemiological studies reporting prevalence and risk factors; (2) compilation of environmental, socioeconomic, and disease burden data from regional databases; and (3) implementation of species distribution modelling algorithms to delineate environmental niches. The models enabled predictive mapping of each disease's ecological niche across Asia based on inferred associations between disease occurrence and environmental conditions.

### Data sources

#### Literature review

We systematically searched major biomedical, regional, and grey literature databases, including PubMed, Scopus, EBSCOhost, Web of Science, Cochrane Library, Cairn, OpenGrey, and Scielo from database inception through December 2018. The search strategy utilized Medical Subject Headings (MeSH) terms including ["*Clonorchis sinensis*", "*Clonorchis sinenses*", "*Clonorchiasis*", "*Opisthorchis sinensis*", "*Opisthorchis sinenses*","Liver fluke"], AND ["Asia", "Epidemiology", "Prevalence"], and relevant variants in [All Fields]. Equivalent subject headings and keywords were used for searches in other databases.

Inclusion criteria were cross-sectional surveys, cohort studies, or case–control studies reporting primary prevalence data or risk factors for clonorchiasis and/or opisthorchiasis in Asia. Studies were required to have laboratory diagnostic testing for infection.

Exclusion criteria were case reports, reviews, opinion pieces, policy documents, animal studies, and studies without primary prevalence data. Two independent reviewers screened all titles, abstracts, and full texts for eligibility. Data on prevalence, diagnostics, location, sample size, demographics, and risk factors were extracted from included studies into a standardized form using Zotero version: 6.0.31 (The Roy Rosenzweig Center for History and New Media, Fairfax, USA). Any discrepancy was resolved by consensus.

This comprehensive literature search aimed to compile all relevant epidemiological data on clonorchiasis and opisthorchiasis prevalence and risk factors needed to inform model development.

### Databases

For *C. sinensis* infection data in the Mekong River region, we supplemented the literature review by compiling primary data on population infection rates from databases in Vietnam and Guangxi Zhuang Autonomous Region of China. Cross-sectional surveys, conducted between 2000 and 2018 in Vietnam, were systematically searched to extract geolocated presence/absence data based on faecal egg detection at the survey point and the regional level. Infection status was coded as Yes (positive) or No (negative) for *C. sinensi* in the databases.

For Guangxi Zhuang Autonomous Region, China, population-level data on *C. sinensis* infections were obtained from the 3rd National Survey on Key Parasitic Diseases conducted between 2014 and 2016, which covered 31 provinces (municipalities, autonomous regions) in rural and urban areas of China. Apart from *C. sinensis*, testing included tapeworms, intestinal protozoa and other key parasitic infections via faecal examination in the sampled population. A stratified cluster sampling method was used, classifying China into 5 endemic zones for *C. sinensis* and sampling within each zone. All individuals in the selected clusters underwent testing. Stool specimens were examined by the Kato-Katz thick smear technique using two smears per specimen to detect intestinal helminth eggs.

By compiling primary epidemiological records from these standardized national surveys in China and Vietnam, we obtained geolocated *C. sinensis* infection data needed to parameterize niche modelling and epidemiological comparisons between the two liver flukes. The original data of *O. viverrini* infection in endemic countries of Southeast Asia is extracted from the 113 studies and combined with the reported data from WHO (Department of Neglected tropical diseases of WHO Western Pacific), and details of system review screening were shown in Additional file 1.

### Environmental data

We compiled 26 natural climatic, and socio-cultural predictor variables, including distance to water bodies, elevation, slope, normalized difference vegetation index (NDVI), land cover, 19 bioclimatic variables (Bio1–Bio19), human influence index (HII), human footprint index (HFP), based on the variables used in Zhao’s study [17], and Zheng’s study[18] for modelling with liver fluke and snails. We additionally included local habit of raw fish consumption-eating as a predictor variable. Our approach was to use a comprehensive set of environmental and socio-economic factors to capture these fine-scale differences. Factors may similarly increase overall risk, but specific values pinpoint geographic boundaries. The machine learning framework integrated with ecological data successfully learned these distinct signatures, enabling accurate discrimination for mapping. All databases used for these 26 predictor variables are shown in Table 1.

**Table 1:** Environmental and climatic variables influencing liver fluke infection

Variables	Descriptions
Water distance (m)	Distance to water bodies
Elevation (m)	Elevation
Slope	Slope
NDVI	Normalized Difference Vegetation Index
Land cover	Land cover
HII	Human influence index
HFP	Human footprint
BIO1 (℃)	Annual mean temperature
BIO2 (℃)	Mean diurnal range (Mean of monthly (max temp–min temp))]
BIO3 (%)	Isothermality (BIO2/BIO7) (× 100)]
BIO4 (%)	Temperature seasonality (standard deviation × 100)]
BIO5 (℃)	Max temperature of warmest month
BIO6 (℃)	Min temperature of coldest month
BIO7 (℃)	Temperature annual range (BIO5-BIO6)
BIO8 (℃)	Mean temperature of wettest quarter
BIO9 (℃)	Mean temperature of driest quarter
BIO10 (℃)	Mean temperature of warmest quarter
BIO11 (mm)	Mean temperature of coldest quarter
BIO12 (mm)	Annual precipitation
BIO13 (mm)	Precipitation of wettest month
BIO14 (mm)	Precipitation of driest month
BIO15	Precipitation seasonality (Coefficient of Variation)
BIO16 (mm)	Precipitation of wettest quarter
BIO17 (mm)	Precipitation of driest quarter
BIO18 (mm)	Precipitation of warmest quarter
BIO19 (mm)	Precipitation of coldest quarter

Topographic variables, such as water distance, elevation, slope, NDVI, and land cover were extracted from the Shuttle Radar Topography Mission (SRTM) at 5 km-resolution (http://srtm.csi.cgiar.org/). Water distance calculates the Euclidean distance from each grid cell to the nearest wetland, including lakes, wetlands, and river floodplains, representing proximity to water bodies (in meters). Elevation denotes altitude above the mean sea level (in meters). Slope describes the rate of change in elevation. NDVI is an index of green vegetation density ranging from -1 to 1, with values below 0 indicating water, cloud, snow; near 0 barren land; and above 0 vegetation cover increasing with density. Land cover was defined using the Moderate Resolution Imaging Spectroradiometer (MODIS) MCD12Q1 product (https://lpdaac.usgs.gov/products/mcd12c1v006/), aggregated and reprojected to match the 15-class University of Maryland scheme.

Geospatial data layers were extracted for each liver fluke survey location to assess environmental factors associated with *C. sinensis* and *O. viverrini* transmission. Univariate comparisons were conducted between survey points for each variable using Mann–Whitney U tests.

Climatic data were obtained from WorldClim v.1.4 at 5 km-resolution (http://www.worldclim.org), interpolated from global weather station data from 1955 to 2000 for China. The 19 bioclimatic variables represent annual trends, seasonality, and limiting factors calculated from monthly temperature and rainfall. These are more biologically meaningful than temperature/rainfall alone.

To represent anthropogenic effects on the environment, we extracted two human influence indices: HII quantifies direct human pressures on ecosystems using population density, built environments, transportation networks, land use/land cover, and nightlights (https://sedac.ciesin.columbia.edu). HII ranges 0–64, with higher values indicating greater human environmental impacts. HFP shows relative human pressure, with red indicating more intense activity.

### Eating habits of raw fish

The study considered the use of data on the consumption of raw fish because eating raw or undercooked fish is a well-known risk factor for infections with *C. sinensis* and *O. viverrini*. These liver flukes can infect humans who consume freshwater fish containing the larval stages of the parasites. This dietary habit directly relates to the transmission dynamics of these parasites, making it a critical factor to examine in understanding the geographical distribution and risk of infection.

By understanding where and how often people consume raw fish, we define the eating habits with reference to raw fish were recorded for mapping sections by provinces and municipalities in all countries based on literature review, coded as 1 if present the eating habits with raw fish or 0 if absent the eating habits with raw fish. Also, we collecting data from affected populations through direct questioning about their dietary habits through the help of local disease control centres and experts from each country as we have consulted with, specifically the dietary habits for consumption of raw or undercooked freshwater fish with specific municipalities areas. Finally, provincial polygons were rasterized to assign presence across each province.

### Assessment and extraction of variable data

The compiled databases were separated into two groups based on human infection with *C. sinensis* and *O. viverrini* for comparative analysis. We statistically summarized and mapped the locations of the two parasite infections. For normally distributed continuous variables, means and standard deviations were calculated, with t-tests used to compare groups. As land cover comprised 15 categorical classes, non-normal variables were summarized using median and interquartile range (IQR) and compared between groups with non-parametric tests.

All data processing and analyses were conducted in R V.4.0.2 (Lucent Technologies, Jasmine Mountain, USA). Variables were assessed for collinearity and eliminated if the variance inflation factor (VIF) exceeded 5. We then used random forest (RF) models to rank predictor importance based on mean decrease in accuracy when excluded from the models. The top 10 most important variables for each parasite were retained for further niche modelling. This process filtered the database variable data to retain only relevant non-redundant predictors characterizing the fundamental and realized niches of the two liver flukes under study. It also allowed statistical comparisons to identify similarities and differences in their ecological and environmental constraints. These ‘curated’ database variable values provide the inputs for ensuing distribution modelling and mapping of the transmission risk.

### Model development

We developed predictive models to classify and discriminate human infections of *O. viverrini* versus *C. sinensis* based on environmental variables, following the framework *Y* = *f(x)*. The binary response variable Y would indicate *O. viverrini* (Y = 0) or *C. sinensis* (Y = 1) infection, the aim was delineating the potential transition zone where the probability for both species shifts between 0 and 1, indicating possible co-endemicity. The predictor variables (*X*) comprised 26 environmental, climatic and socio-cultural factors. To account for class imbalance, we used the SMOTE algorithm from the *DMwR* package to synthesize additional minority class examples. The models were constructed and evaluated using the Caret package in R. To enable consistent comparison across algorithms and assessment of variable importance, we selected six commonly used machine-learning classification methods to model environmental suitability for *O. viverrini* and *C. sinensis* transmission: linear regression (LM), decision trees (DT), neural networks (NNET), RF, gradient boosting machines (GBM) and extreme gradient boosting (XGBOOST). Details on each algorithm can be found at the Caret documentation (https://topepo.github.io/caret/index.html). All models were trained using tenfold cross-validation repeated 5 times, with hyperparameter tuning to optimize model performance. Model fitting performance, prediction accuracy, variable contributions, marginal response plots, and projected distribution maps were analysed and evaluated for each approach.

The fitted machine learning models were applied to an independent testing dataset to evaluate generalizability. Liver fluke presence/absence predictions were generated for each testing location and compared to observed outcomes to assess model discrimination. Testing performance was quantified using AUC, accuracy, Kappa value, sensitivity, and specificity metrics.

We evaluated and compared models based on the area under the receiver operating characteristic curve (AUC) by sensitivity, specificity, and Cohen's Kappa statistic. The optimal model was selected based on having the highest cross-validated AUC. This model was then finalized by refitting on the full dataset to generate the final prediction equation.

Model development aimed to maximize discrimination accuracy in predicting *O. viverrini* versus *C. sinensis* infections based on ecological and environmental factors relevant to their transmission dynamics and geographic distributions. The resulting model could then be applied to mapping transmission risk and predicting changes under climate change scenarios. Model development and validation followed a rigorous workflow for tuning, testing and application. The compiled database of values for the two infections was randomly split into a training set (70% of the data) for model calibration and a testing set (30% of the data) for independent evaluation. The training data underwent fivefold cross-validation, whereby the data were divided into 5 equal partitions. In each fold, models were fitted on 4 partitions and predictions generated for the held-out fold. This process was repeated, holding out each partition in turn to identify the optimal hyperparameters that minimized the cross-validation error. This was done as cross-validation prevents model overfitting and provides a realistic estimate of performance on new data.

Following cross-validation-based tuning, the final models were refit on the full training set using optimal hyperparameters. Model skill was quantified on the training set using the AUC as mentioned above. The tuned models were then applied to the previously held-out testing set to evaluate performance on new data. Variable importance was calculated by excluding each predictor and quantifying loss in testing AUC. Marginal effects of key predictors were generated from the finalized models to quantify variable-outcome relationships. Model predictions were mapped across the study region based on environmental inputs to predict risk areas for each species. Finally, an ensemble approach was taken by integrating predictions across algorithms to leverage model strengths.

### Model assessment and prediction

Model calibration was assessed using calibration plots to evaluate agreement between predicted and observed outcomes. Classification metrics including AUC, accuracy, Kappa value, specificity, and sensitivity were calculated at the optimal probability threshold to quantify model discrimination ability. Variable importance was determined using the *varImp* function in the Caret package, which quantifies the decrease in model AUC with variable exclusion. This approach includes all predictors and ranks importance based on change in performance. Marginal effects of key variables were visualized using partial dependence plots (PDPs) from the *pdp* package. PDPs show the functional relationship between a predictor and the outcome while accounting for effects of other variables. To reduce computation time, PDPs were generated for the top three important variables.

The finalized models were applied to predict the probability of *C. sinensis* infection across gridded environmental data in China's Guangxi Zhuang Autonomous Region and the south-eastern Laos, Thailand, Cambodia, and Vietnam Regions. Predictions were mapped to visualize the geographic distribution of estimated risk. Any predictions of *C. sinensis* in Guangxi Zhuang Autonomous Region of China were considered erroneous given known distributions. To delineate species boundaries, we focused on areas of Vietnam and Laos where both species are endemic. Grid cells with a predicted probability of *C. sinensis* of 100% were classified as high risk for that species. Areas with intermediate probabilities of 0–1 were considered potential hybrid zones with sympatric transmission.

## Results

### Geographic distribution

**Fig. 1:**
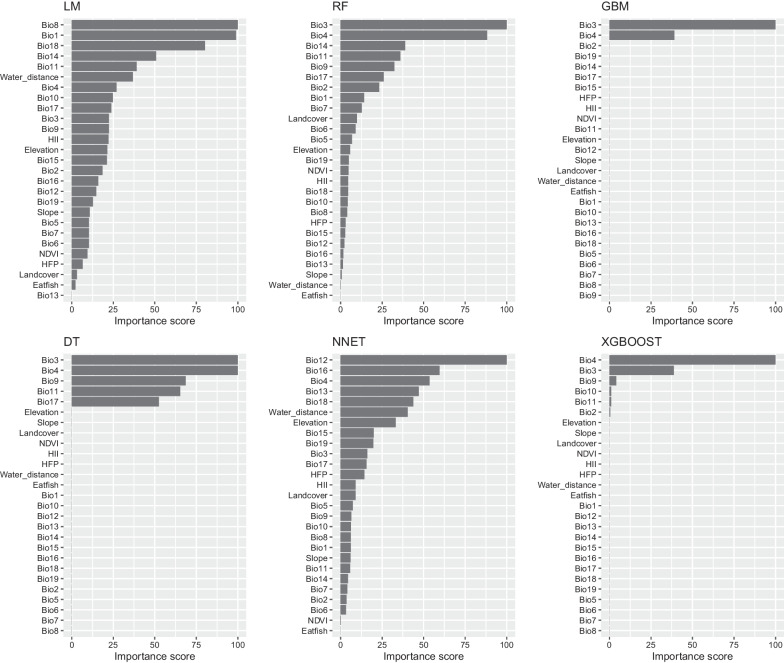
Feature Importance Analysis in *Clonorchis sinensis* and *Opisthorchis viverrini* infections of six predictive models (The models displayed are Linear Model (LM), Random Forest (RF), Gradient Boosting Machine (GBM), Decision Tree (DT), Neural Network (NNET), and eXtreme Gradient Boosting (XGBOOST). Each bar represents the importance score of ecological and environmental features, such as Bioclimatic variables (Bio1-Bio19), elevation, slope, land cover, NDVI (Normalized Difference Vegetation Index), HII (Human Influence Index), HFP (Human Footprint), and distance to water bodies)

This study found the Southeast Asian range of *O. viverrini* and *C. sinensis* infections to be cantered in China, Vietnam, Thailand, Laos, and Cambodia (Prediction map was shown Figure1.png in https://github.com/jamesjin63/Liver_fluke/). In the study period (2000–2018), *C. sinensis* infections were identified in 84 places extracted from data in 15 studies, with a concentration in southern China (Guangxi Zhuang Autonomous Region) and northern Vietnam. In contrast, *O. viverrini* infections were seen in 721 places, extracted from 113 studies, with clusters appearing across Thailand, Laos, Cambodia, and Vietnam. These spatial patterns align with known endemic zones based on past national surveys and reported cases. However, our database compiled a larger number of geocoded epidemiological studies that provided mapping with higher resolution of local prevalence.

### Environmental associations

Univariate comparisons conducted between survey points for elevation variable showed median elevation to be significantly lower in *C. sinensis* locations (median = 35.44 m, IQR: 12.2–98.7) compared to *O. viverrini* ones (median = 159.5 m, IQR: 45.6–212.4; *P* < 0.0001). No significant differences were found for slope, Bio10, Bio8, or Bio16 (See Table 2).

**Table 2:** Comparative ecological analysis of *Clonorchis sinensis* and *Opisthorchis viverrini* infections of environmental, climatic, and socio-economic variables

Variables	Liver fluke		*P* value
	*C. sinensis* (*N* = 84)	*O. viverrini* (*N* = 721)	
Elevation*	35.44 (3.12, 177.00)	160.00 (108.13, 238.37)	< 0.001
Slope*	0.73 (0.14, 4.80)	0.62 (0.32, 1.68)	0.593
Land cover*	12.00 (9.00, 12.00)	12.00 (8.00, 12.00)	0.003
NDVI*	0.46 (0.37, 0.65)	0.53 (0.42, 0.70)	0.003
HII	29.50 ± 9.36	22.00 ± 7.61	< 0.001
HFP	45.98 ± 14.57	33.77 ± 11.58	< 0.001
Water distance*	1033.20 (168.53, 5872.07)	3925.45 (544.82, 8706.90)	0.002
Bio1	22.35 ± 1.70	26.06 ± 1.78	< 0.001
Bio2	6.67 ± 0.83	9.78 ± 1.30	< 0.001
Bio3	32.46 ± 2.26	55.21 ± 3.77	< 0.001
Bio4	522.83 ± 85.88	215.00 ± 53.04	< 0.001
Bio5	31.90 ± 1.10	34.19 ± 2.28	< 0.001
Bio6	11.28 ± 3.22	16.39 ± 2.65	< 0.001
Bio7	20.62 ± 2.93	17.80 ± 2.67	< 0.001
Bio8	27.27 ± 1.73	26.97 ± 1.33	0.052
Bio9	16.20 ± 2.12	23.36 ± 2.40	< 0.001
Bio10	28.14 ± 1.14	28.34 ± 1.61	0.269
Bio11	15.48 ± 2.71	23.07 ± 2.31	< 0.001
Bio12	1684.77 ± 150.96	1566.70 ± 470.23	0.022
Bio13	338.17 ± 51.37	332.66 ± 131.55	0.704
Bio14	26.02 ± 9.87	4.40 ± 5.27	< 0.001
Bio15	80.11 ± 8.22	89.44 ± 9.70	< 0.001
Bio16	899.33 ± 111.56	858.59 ± 335.65	0.27
Bio17*	95.83 (78.34, 112.29)	20.50 (17.91, 27.72)	< 0.001
Bio18*	772.30 (736.63, 820.49)	367.71 (289.75, 534.85)	< 0.001
Bio19	105.08 ± 38.85	49.31 ± 55.53	< 0.001

Continuous data are presented as mean ± standard deviation; *represents the median (interquartile range, IQR with Q1,Q3); Elevation, Height above sea level, in meters (m); Slope, Steepness or incline of land, in degrees (°); NDVI, normalized difference vegetation index; Land cover, Type of land cover, categorized; HII, human influence index; HFP, human footprint index; Water distance, Distance to nearest water body, in meters (m); BIO1, annual mean temperature (°C); BIO2, mean diurnal temperature range (°C); BIO3, temperature annual range (% of mean); BIO4, temperature seasonality (standard deviation); BIO5, maximum temperature of the warmest month (°C); BIO6, minimum temperature of the coldest month (°C); BIO7, annual temperature range (°C); BIO8, mean temperature of the wettest quarter (°C); BIO9, mean temperature of the driest quarter (°C); BIO10, mean temperature of the warmest quarter (°C); BIO11, mean temperature of the coldest quarter (°C); BIO12, annual precipitation (mm); BIO13, precipitation of the wettest month (mm); BIO14, precipitation of the driest month (mm); BIO15, precipitation seasonality (coefficient of variation); BIO16, precipitation of the wettest quarter (mm); BIO17, precipitation of the driest quarter (mm); BIO18, precipitation of the warmest quarter (mm); BIO19, precipitation of the coldest quarter (mm)

### Model fitting with training data

Based on the compiled training dataset, the six algorithms learned associations between environmental predictors and liver fluke presence/absence to develop fitted models. All models achieved excellent fitting performance on the training data with AUC, accuracy, Kappa value, sensitivity, and specificity approaching 1 (NNET range: 0.981–0.998). (Table 3).

**Table 3:** Training model fit metrics for the machine learning approaches

Model	AUC	Threshold	Accuracy	Kappa	Sensitivity	Specificity
LM	1	0.093	1	1	1	1
RF	1	0.093	1	1	1	1
GBM	1	0.093	1	1	1	1
DT	1	0.093	1	1	1	1
NNET	0.996	0.093	0.991	0.998	0.981	1
XGBOOST	1	0.091	1	1	1	1

AUC, area under the receiver operating characteristic curve; Threshold, optimal probability threshold for model predictions; Accuracy, overall accuracy of model predictions; Kappa, Cohen's Kappa statistic measuring prediction agreement; Sensitivity, model sensitivity in predicting presence; Specificity, model specificity in predicting absence; RF, random forest model; XGBOOST, extreme gradient boosting model; GBM, gradient boosting machine model; LM, logistic regression model; DT, decision tree model; NNET, neural network model

### Model predictions on independent testing data

All models achieved excellent prediction accuracy on the testing data with AUC, accuracy, kappa value, sensitivity, and specificity approaching 1 (NNET range: 0.991–0.996) (Table 4).

**Table 4:** Parameters of model performance in the testing set

Model	AUC	Threshold	Accuracy	Kappa	Sensitivity	Specificity
LM	1	0.117	1	1	1	1
RF	1	0.117	1	1	1	1
GBM	1	0.117	1	1	1	1
DT	1	0.117	1	1	1	1
NNET	0.991	0.117	0.999	0.996	0.9964	1
XGBOOST	1	0.117	1	1	1	1

AUC, area under the receiver operating characteristic curve; Threshold, optimal probability threshold for model predictions; Accuracy, overall accuracy of model predictions; Kappa, Cohen's Kappa statistic measuring prediction agreement; Sensitivity, model sensitivity in predicting presence; Specificity, model specificity in predicting absence; RF, random forest model; XGBOOST, extreme gradient boosting model; GBM, gradient boosting machine model; LM, logistic regression model; DT, decision tree model; NNET, neural network model

### Key environmental drivers

The machine-learning models identified Bio4 and Bio3 as consistently influential predictors of liver fluke presence across techniques (Fig. 1), agreeing with their known role in snail habitat suitability.

### Marginal variable effects

**Fig. 2:**
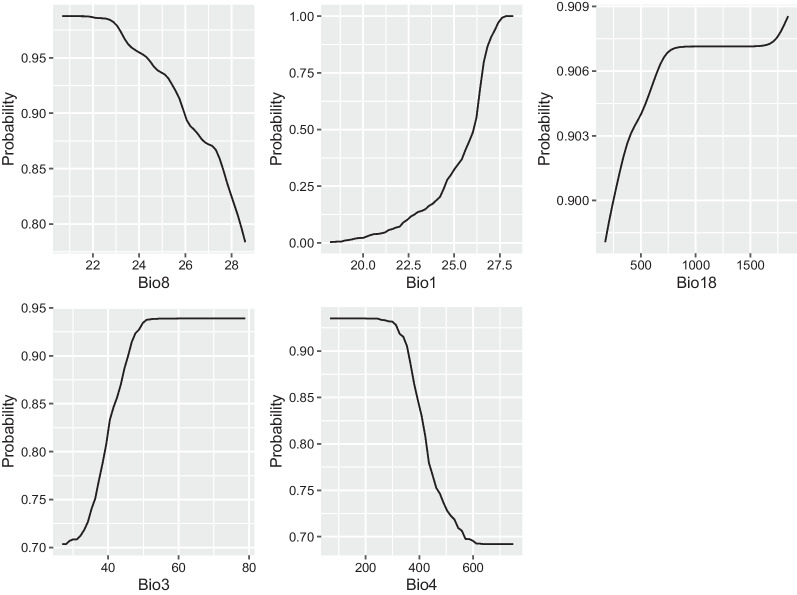
Partial dependence plots (PDPs) for *Clonorchis sinensis* (Y = 1) infections of RF model predictive (The figure consists of PDPs that depict the relationship between selected Bioclimatic variables (Bio1, Bio3, Bio4, Bio8, Bio18) and the probability of *Clonorchis sinensis* infection as predicted by RF model. Each plot shows how changes in a specific Bioclimatic variable impact the model's predicted probability of infection, holding all other features constant)

Based on the fitting and prediction results, all models except NNET showed good performance. Using the LM model, the variables Bio8, Bio1, and Bio18 were selected for partial dependence plots based on contribution over 75%. For the RF model, Bio4 and Bio3 were selected. The plots visualize the dependence between variables and predicted probability of liver fluke of *C. sinensis* presence (Y = 1). From Fig. 2, results showed Bio8 had a predicted probability of 0.987 at 22 °C, decreasing as Bio8 increased. Probability of presence increased with Bio1, with values below 0.1 when Bio1 = 22.4 °C and reaching 1 when Bio1 > 27 °C. For Bio18, predicted probability was above 0.87, increasing to over 0.9 when Bio18 exceeded 750 mm. Bio3 exhibited increasing predicted probability with values, with plateau creation at 0.93 when Bio3 > 50. In contrast, as Bio4 increased, predicted probability declined. These patterns align with known biological requirements of snail intermediate hosts and transmission potential. The marginal response plots aid interpretation of complex model dynamics.

### Model predictions

The fitted machine-learning models were used to estimate the geographic distribution of liver fluke infection risk across the Mekong River basin and Guangxi Zhuang Autonomous Region of China (Prediction of liver fluke infection risk map was shown Figure 2.png in https://github.com/jamesjin63/Liver_fluke/). Each model generated a predicted probability surface for liver fluke presence at each location based on the input variables. All models successfully delineated areas of higher predicted risk for *C. sinensis* versus *O. viverrini*. However, the NNET and RF models projected some *C. sinensis* presence in northern Vietnam and southern China (Guangxi Zhuang Autonomous Region). The DT, GBM, and XGBOOST models limited *O. viverrini* suitability to western Guangxi Zhuang Autonomous Region of China, while the LM model predicted only *C. sinensis* across the all Guangxi Zhuang Autonomous Region of China.

### Delineation of boundaries

To further investigate the geographic boundaries between *C. sinensis* and *O. viverrini*, the study area was narrowed to Vietnam and Laos. Of the model predictions, only the RF results met all simulated conditions for delineating species risk. The RF model generated a predicted risk map for *C. sinensis* and *O. viverrini* in Vietnam and Laos (Prediction map was shown Figure 3.png in https://github.com/jamesjin63/Liver_fluke/). Three types of regions have been classified in Figure 3.png, including: Region A (in red) indicated a higher predicted risk for *O. viverrini* transmission, with a focus in central and southern Vietnam plus few northern provinces of Vietnam bordering Laos, as well as whole areas of Laos. Region B (in blue) depicted higher *C. sinensis* transmission risk in northestern Vietnam, adjoining the border to Guangxi Zhuang Autonomous Region of China. Region C (in pink) represented a transitional zone of mixed species potential, where the RF model predicted *O. viverrini* transmission probability < 1. This spanned 4 northwestern provinces and 2 northcentral provinces of Vietnam, as well as small areas in northern Laos bordering Vietnam.

## Discussion

Of the six machine-learning models developed in this study, all except the NNET model, accurately classified and predicted *C. sinensis* and *O. viverrini* infections during model fitting and projection across the Mekong River Basin and Guangxi Zhuang Automomous Region of China. Despite known absence of *O.viverrini* in China, the RF model predicted potential presence in small areas of northwestern Guangxi Zhuang Autonomous Region of China, either indicating inaccurate classification or undiscovered transmission. The LM model predicted no any *O. viverrini* transmission risk in China, but modelled suitable areas across central and southern Vietnam, aligning with national surveys [19, 20]. Reportedly endemic areas across 21 northern provinces of Vietnam for *C. sinensis* infections, concentrated near the Red River Basin [14]. *O. viverrini* persists across 11 central provinces of Vietnam while control efforts have eliminated southern foci there [21]. However, complex co-endemic areas remain understudied, with no reports from north-western Vietnam [5]. Sithithaworn et al. previously delimited a diagonal boundary from Lai Chau Province northwest to Quang Binh Province central-east of Vietnam, designating upper and lower zones for *C. sinensis* and *O. viverrini*, respectively [20, 22]. We identified a transition zone of mixed transmission risk in Vietnam, with suitable environments for both flukes, spanning four northwestern provinces and two northcentral provinces. Visualizing the projected ranges advances an understanding of potential overlapping. As these infections depend on human culinary practices, mixed zones likely reflect localized food habits including raw fish dishes.

The distinct geographic distributions of *C. sinensis* and *O. viverrini* motivated an analysis of geographic, climatic, and anthropogenic predictors, revealing divergence between the two flukes. For example, the former occurred mostly in low latitudes while the latter predominated in higher latitudes. In Thailand, *O. viverrini* is concentrated in the Northeast with a similar high latitude pattern in Laos [23]. Most georeferenced *O. viverrini* occurrences are from Thailand. Reports from Vietnam noted *C. sinensis* as concentrated around the Red River Basin in lower latitudes [24]. Climate also differed between endemic areas, with mean annual precipitation of 772 mm for *C. sinensis* versus 367 mm for *O. viverrini*. These factors likely influenced fluke distributions indirectly by impacting snail intermediate hosts. A study in Thailand found *O. viverrini* sensitive to rainfall and minimum temperature, with consistent prevalence from 41–356 mm monthly rainfall but a drop above 23 °C [25]. The most influential predictors varied among models constructed here. Considering variables contributing over 75% for *O. viverrini*, key factors were annual mean temperature (Bio1), temperature seasonality (Bio4), warmth index (Bio8), rainfall in warmest quarter (Bio18) and annual temperature range (Bio3). The LM dependency plot showed *O. viverrini* probability increasing with temperature seasonality. Infection likelihood peaked around 300% variance in seasonal temperatures and with over 750 mm precipitation in the warmest quarter. As annual mean temperature rose above 27 °C, *O. viverrini* probability approached 100%. These results highlight climatic factors, especially temperature and rainfall, as important delineators between *O. viverrini* and *C. sinensis* distributions. In differentiating the distribution of *O. viverrini* and *C. sinensis*, our study identified several critical influencing factors. For *C. sinensis*, factors such as higher temperatures and urbanized environments showed greater association, whereas *O. viverrini* distribution was more influenced by wetland ecosystems and certain agricultural practices, and lower elevation ranges for the *Bithynia* spp. snail hosts of *O. viverrine* [26]. The distinction in habitat preferences, intermediate host snail species, and human behaviours, including dietary differences across regions, were significant in delineating the distribution of these flukes. These variables were crucial in our machine learning models to predict geospatial distribution with greater specificity.

The results demonstrated environmental and climatic variables shape distributions of *C. sinensis* and *O. viverrini*. Divergence across factors enables classification, with models accurately categorizing infections in test data. However, as Max Kuhn notes, machine-learning risks finding spurious relationships if predictors closely parallel outcomes, producing apparent 100% accuracy for uninformative variables. While dividing flukes using single factors proved successful here, disease emergence involves complex interactions among environmental, climatic, and social determinants [27]. Despite ideal performance during training, GBM leaning solely on annual mean temperature range and temperature seasonality generated some geographically discordant projections. This highlights the need to validate models against real-world data, not just internal fit, when applying predictions. While these spatial models’ further knowledge of potential *C. sinensis* and *O. viverrini* distributions, multifaceted drivers and potential sampling biases warrant caution for public health planning until localized surveys confirm patterns. Elimination efforts require understanding mixed-disease contexts through on-the-ground investigation. Our models successfully distinguished northern endemic areas for *C. sinensis* from southern *O. viverrini* foci but also delimited a transitional zone of overlapping potential spanning 6 north-western Vietnamese provinces where both liver flukes may persist. This coexistence complicates control efforts designed for single infection. The policy recommendation should be with a multi-pronged approach in these zones of sympatry, incorporating coordinated interventions tailored to each species while integrating education and policy to maximize efficacy. This includes dual drug administration with praziquantel and tribendimidine to target both flukes, augmented diagnostics to distinguish infections, ecological modifications limiting snail intermediate hosts, and sociocultural promotion of cooked fish consumption given dietary habits underlying transmission. Robust surveillance is vital to monitor efforts [28].

Although this study collected georeferenced human infections to classify liver flukes using environmental predictors and machine-learning, limitations include reliance on reported occurrence points from prior literature, lack of animal infection data, exclusion of intermediate snail/fish host distributions, assumptions that all suitable environments have active transmission, and generalizability constraints of machine learning algorithms. Additionally, while we identified sympatric zones, molecular evidence would further confirm co-endemicity. Future work should incorporate such data to delineate ranges. Our ecological approach provided initial delineation in Vietnam, but molecular epidemiology is needed to confirm potential boundaries or zones of sympatry. Further studies should identify underlying drivers, be it intermediate host compatibility or fluke biology. Questions remain whether a sharp boundary exists and what drives separation. To address these complexities, a multidisciplinary collaboration that synthesizes eco-epidemiology, molecular epidemiology, malacology, and parasitology is essential. This would allow for a deeper understanding of fluke ecology, inform targeted control programs, and support the global effort to combat these neglected tropical diseases within the framework of the One Health concept [29].

## Conclusions

This study delineated the boundary between *C. sinensis* and *O. viverrini* in the Mekong River Basin, identifying sympatric transmission in Vietnam concentrated in northwestern and northcentral provinces, and part of northern Laos. Environmental, climatic, and sociocultural factors diverged between the endemic areas, with rainfall in the warmest quarter, precipitation in the wettest month and annual mean temperature influencing distributions most. Machine-learning models effectively classified the endemic areas of liver flukes, demonstrating utility for mapping boundaries. Elimination of these neglected tropical diseases requires understanding the mosaic of species and targeting control and surveillance to local transmission patterns. Further molecular epidemiological studies can confirm the potential boundary and drivers shaping this divergence across Southeast Asia.

### Supplementary Information

**Additional file 1. **The process of extracting data from public database.

## Data Availability

The datasets generated and/or analyzed during the current study are available from the corresponding author on reasonable request.

## Abbreviations

FBTs: Food-borne trematodiases
NTDs: Neglected tropical diseases
AUC: Area under the curve
RF: Random forest
GBM: Gradient boosting machines
XGBOOST: eXtreme gradient boosting
NNET: Neural networks
LM: Linear model
DT: Decision trees
MeSH: Medical subject headings
NDVI: Normalized difference vegetation index
HII: Human influence index
HFP: Human footprint index
SRTM: Shuttle radar topography mission
MODIS: Moderate resolution imaging spectroradiometer
VIF: Variance inflation factor
SMOTE: Synthetic minority over-sampling technique
