# Supplementary material for: Natural variables separate the endemic areas of Clonorchis sinensis and Opisthorchis viverrini along a continuous, straight zone in Southeast Asia
Source: Infect Dis Poverty. 2024 Mar 12;13:24. doi: 10.1186/s40249-024-01191-7 (PMC10935802; doi:10.1186/s40249-024-01191-7)
Supplement: Supplementary file 1 — Additional file 1. The process of extracting data from public database. [file 40249_2024_1191_MOESM1_ESM.docx]

Supplementary File 1. systematic review searching for live fluke database.

1*. Clonorchis sinensis* infection data

We collected relevant publications reporting prevalence data of clonorchiasis in Lao PDR and China through a systematic review according to the PRISMA guidelines. We searched PubMed and ISI Web of Science from inception to December 31, 2018, with search terms: ((Clonorchis sinensis) OR (liver fluke)) AND ((Lao PDR) OR (China)). We set no limitations on language, date of survey, or study design in our search strategy. For literatures not found by the above methods, we also reviewed reports from governments or Ministry of Health, theses, relevant books, and documents. And this is the flowchart for literature search process. Finally, we gathered the Geo-location data from both system review and WHO (Department of Neglected tropical diseases of WHO Western Pacific) reported data.


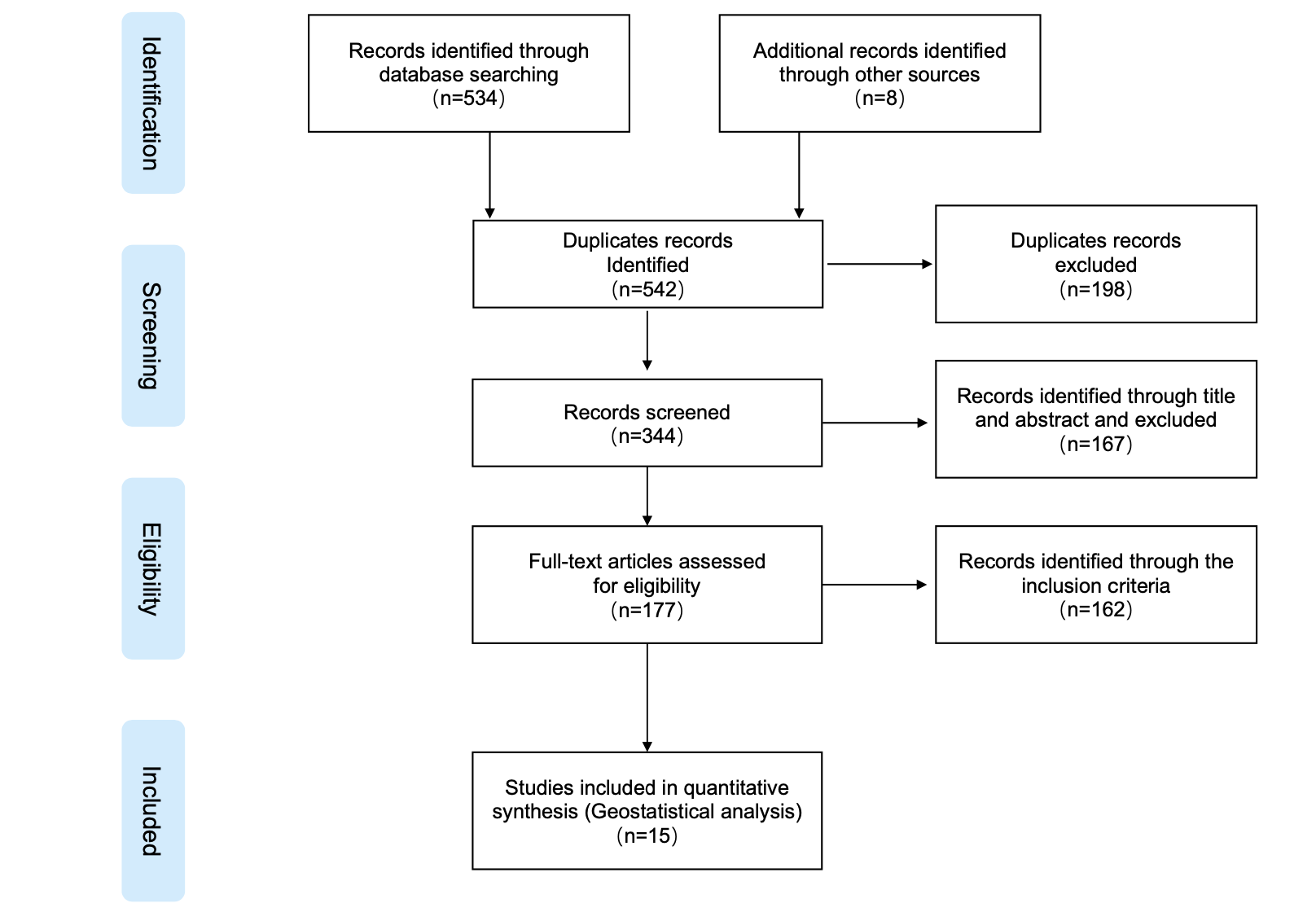


#### Figure S1 Flowchart for literature search on of the *Clonorchis sinensis*

2*. O. viverrini* infection data

Form this part, we collected relevant publications reporting prevalence data of opisthorchiasis in southeast Asia through a systematic review according to the PRISMA guidelines. We searched PubMed and ISI Web of Science from inception to December 31, 2018, with search terms: (liver fluke OR Opisthorchi*) AND (Southeast Asia OR (Myanmar OR Burma) OR Thailand OR Vietnam OR Lao PDR OR Cambodia). We set no limitations on language, date of survey, or study design in our search strategy. For literatures not found by the above methods, we also reviewed reports from governments or Ministry of Health, theses, relevant books, and documents. And this is the flowchart for literature search process. Finally, we gathered the Geo-location data from both system review and WHO (Department of Neglected tropical diseases of WHO Western Pacific) reported data.


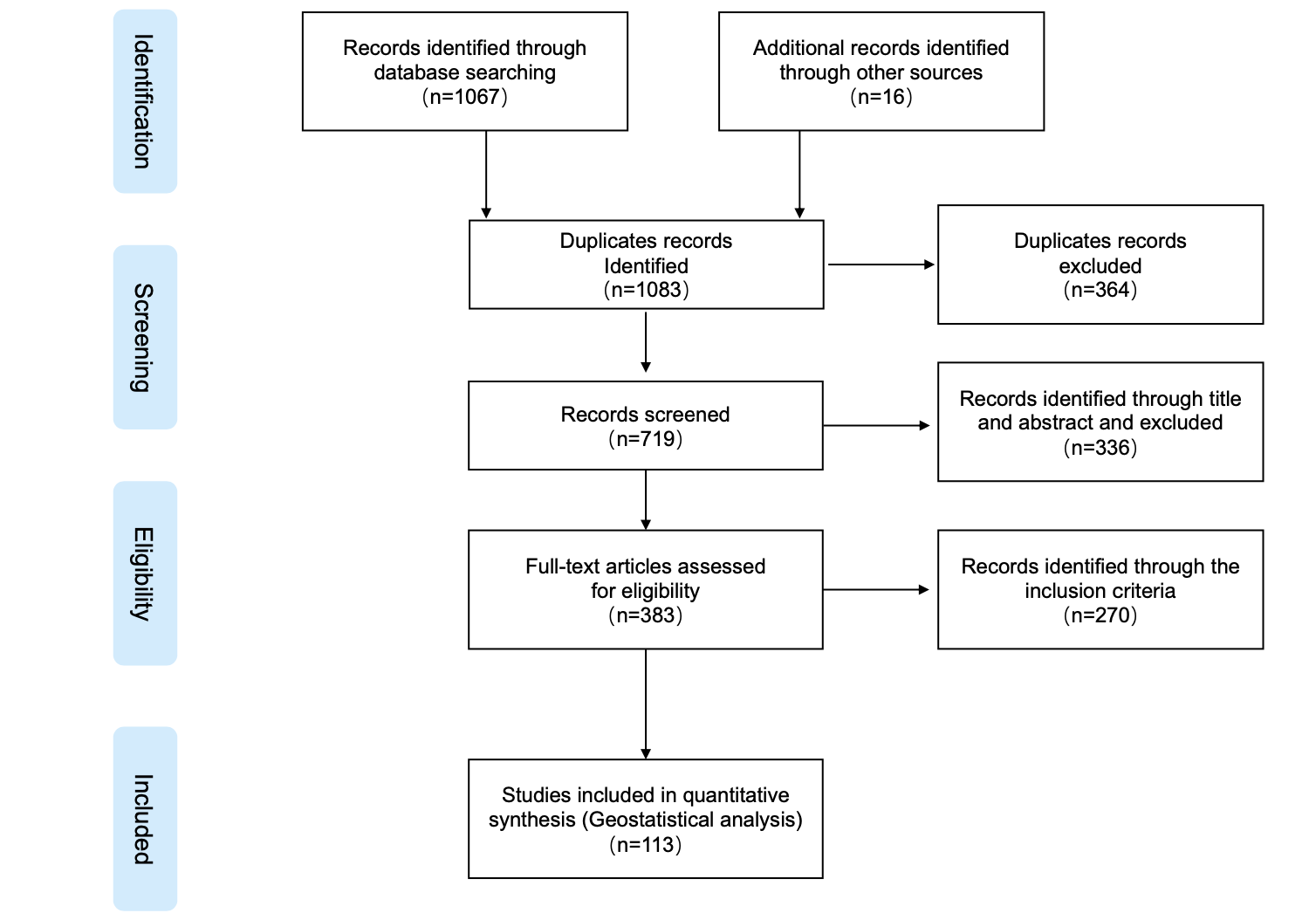


#### Figure S2 Flowchart for literature search on of the *O. viverrini*

The figures of the map were shown in:

https://github.com/jamesjin63/Liver_fluke/
